# Designed nitrogen doping of few-layer graphene functionalized by selective oxygenic groups

**DOI:** 10.1186/1556-276X-9-646

**Published:** 2014-11-29

**Authors:** Ying Chen, Bingqiao Xie, Yingtao Ren, Mengying Yu, Yang Qu, Ting Xie, Yong Zhang, Yucheng Wu

**Affiliations:** 1 Engineering Research Center of Nano-Geomaterials of Ministry of Education, China University of Geosciences, Wuhan 388 Lumo RD, Wuhan 430074, China; 2 Zhejiang Research Institute, China University of Geosciences, Wuhan 388 Lumo RD, Wuhan 430074, China; 3 School of Materials Science and Engineering, Hefei University of Technology, Hefei 230009, China; 4 Key Laboratory of Advanced Functional Materials and Devices of Anhui Province, Hefei 230009, China

**Keywords:** Few-layer graphene, Oxygenic functional groups, Nitrogen doping, Hydrothermal

## Abstract

Few-layer nitrogen doped graphene was synthesized originating from graphene oxide functionalized by selective oxygenic functional groups (hydroxyl, carbonyl, carboxyl etc.) under hydrothermal conditions, respectively. Transmission electron microscopy (TEM) and atomic force microscopy (AFM) observation evidenced few-layer feature of the graphene oxide. X-ray diffraction (XRD) pattern confirmed phase structure of the graphene oxide and reduced graphene oxide. Nitrogen doping content and bonding configuration of the graphene was determined by X-ray photoelectron spectroscopy (XPS), which indicated that different oxygenic functional groups were evidently different in affecting the nitrogen doping process. Compared with other oxygenic groups, carboxyl group played a crucial role in the initial stage of nitrogen doping while hydroxyls exhibited more evident contribution to the doping process in the late stage of the reaction. Formation of graphitic-like nitrogen species was controlled by a synergistic effect of the involved oxygenic groups (e.g., -COOH, -OH, C-O-C, etc.). The doping mechanism of nitrogen in the graphene was scrutinized. The research in this work may not only contribute to the fundamental understandings of nitrogen doping within graphene but promote the development of producing novel graphene-based devices with designed surface functionalization.

## Background

Nitrogen doping in graphene has proven to be an effective way in tailoring properties of pristine graphene and extending its applications [1-6] because doped nitrogen atoms could modify surface chemical state, crystal structure, as well as electronic structure of graphene. Due to great potential of nitrogen-doped graphene (N-doped graphene) in various applications such as catalysis, lithium-ion batteries, supercapacitors, fuel cells, hydrogen storage materials, etc. [3], it is for granted that synthesis and doping mechanism of N-doped graphene with high N content and designed nitrogen configuration need more attention from the view of structure and property control.

Currently, techniques for growing graphene mainly include chemical vapor deposition, arc-discharge, thermal annealing, and solvothermal method [4-8]. NH_3_ has been an important nitrogen source in doping graphene. For example, N-doped graphene could be obtained by annealing GO in NH_3_ atmosphere, and temperature affected the N-doped type, degree of reduction, and N-doping [9,10]. Compared to NH_3_, urea is less corrosive and hazardous and is facile to handle. By replacing NH_3_ with urea, low N content N-doped graphene with pyridinic and pyrrolic N species was obtained at low temperature, while high N content N-doped graphene with graphitic N species was synthesized at high temperature, showing analogous doping results to the case using NH_3_. N-doped graphene also could be synthesized under hydrothermal conditions, and the degree of reduction and N content of graphene was dependent on the amount of urea [11-14].

To the best of our knowledge, previous reports were mainly focused on the effect of experimental parameter changes (temperature, ratio of chemical agents, reaction time) on the nitrogen doping within graphene, and few investigation has been conducted on the influence of oxygenic groups on the graphene surface which may be a non-trivial factor in controlling nitrogen doping process.

Graphene oxide has been reported as an intermediate during graphene synthesis by chemical method [15,16]. It has abundant functional groups such as epoxide, hydroxyl, phenol carbonyl, and lactone on its basal planes and edges [17], which are often used as precursor for preparing graphene composites or modified graphene [18,19]. Due to different reaction activity among oxygenic groups, functionalizing graphene by selective oxygenic groups may impact content and bonding configuration of nitrogen on the graphene surface [20-22].

In this work, we have synthesized various graphene oxides with hydroxyl, carbonyl, and carboxyl groups and reduced the graphene oxide using urea under hydrothermal condition. N-doped graphene with different content and bonding configuration of nitrogen was achieved (N content up to 9.5 at.%). The result indicated that carboxyl groups played the main role in the initial stage of nitrogen doping into graphene while hydroxyls were subsequently involved and promoted the nitrogen doping in the late stage of the reaction. It was found that graphitic-like N species was therein the most difficult N species to be obtained, whose formation could be ascribed to synergistic reactions of different oxygenic groups (e.g., -COOH, -OH, C-O-C, etc.) and also correlated to the distribution and position of the oxygenic groups.

## Methods

### Materials

Following chemicals were chosen for the experiments: natural flake graphite (graphite, purity 99.8%, 600 meshes, Qingdao, China), KMnO_4_ (AR, Sinopharm Chemical Reagent Co., Ltd., Shanghai, China), H_2_NCONH_2_ (99%, AR, Tianjin Kaitong Chemical Reagent Co., Ltd., Tianjin, China), H_2_SO_4_ (98%, AR, Xinyang Chemical Reagent Factory, Xinyang, China), H_3_PO_4_ (85%, AR, Sinopharm Chemical Reagent Co., Ltd., Shanghai, China), HCl (37%, Sinopharm Chemical Reagent Co., Ltd., Shanghai, China), H_2_O_2_ (30%, Sinopharm Chemical Reagent Co., Ltd., Shanghai, China), C_2_H_5_OH (Sinopharm Chemical Reagent Co., Ltd., Shanghai, China), ClCH_2_CO_2_H (Shanghai Taishan Chemical Plant, Shanghai, China), NH_3_ • H_2_O (30%), home-made secondary deionized water. All chemicals were not treated with further purification.

### Characterization methods

Morphology, composition, microstructure, and surface chemistry of the samples were characterized by JEOL2010 transmission electron microscope (TEM, accelerating voltage of 200 KV, JEOL, Akishima-shi, Tokyo, Japan), PHI-5400 X-ray photoelectron spectroscopy (XPS), X-ray diffraction (XRD), and Nicolet-Nexus Fourier transform infrared spectroscopy (FT-IR).

### Experimental process

#### Synthesis of Graphene oxide (GO)

Three grams of natural flake graphite was added into 400 ml of ice-mixed acid (H_2_SO_4_:H_3_PO_4_ = 9:1 in volume). Then, 18 g of KMnO_4_ was slowly added. After the temperature was heated to and kept at 50°C, the solution was stirred for 60 h. Finally, the solution was cooled down to room temperature. Deionized water of 300 ml was slowly added for dilution, and 10 ml of H_2_O_2_ and 100 ml of HCl were added to remove impurities. Then, a large amount of distilled water was used to make washing and centrifugation, which was repeated for several times until the pH of the solution was adjusted to 5. The brown precipitate was frozen and dried for preservation.

### Synthesis of GO-OOH

A 0.5 g of GO was dispersed into 250 ml deionized water to obtain a transparent yellow solution. Then, 10 g of NaOH and 7.5 g of ClCH_2_COOH were added to the solution under ultrasonic treatment for 3 h. Then, the sample was centrifugated and washed by a large amount of deionized water for several times until the pH of the solution was neutral. Finally, the black precipitate was dried under vacuum at 60°C.

### Synthesis of Ge-OH

A 180 mg of graphene obtained from a thermal treatment on GO (1,000°C for 30 s) was dissolved into a 20.4 ml methylbenzene. After an ultrasonic treatment for 45 min, the solution was washed with pentane and filtrated to obtain a black solid. Then, 105 mg of the black solid was dispersed into 35.1 ml of deionized water again by adding 52.5 mg of FeSO_4_ · 7 H_2_O and 10.5 ml of H_2_O_2_ (30%). A well-dispersed solution was obtained after stirring for 12 h at room temperature. Centrifugal washing was then performed for several times with deionized water to remove the contaminants. Finally, the sample was dried under vacuum at 60°C.

### Synthesis of GO = O

Process for preparation is the same as Ge-OH, except that the precursor used in this system is GO.

### Synthesis of GO-avg

A 0.3 g of GO was dispersed into 150 ml deionized water by adding 36 g of NaOH and 30 g of ClCH_2_COOH. Then, ultrasonic reaction was performed for 1.5 h under controlled temperature (approximately 10°C) for another 1 h. Then, the mixture was washed with deionized water and centrifuged for several times until the pH was adjusted to 5. The precipitate was dried under vacuum at 60°C for preservation.

#### Synthesis of nitrogen-doped graphene

Twenty-five milliliters of GO (GO-OH, GO = O, GO-OOH, or GO-avg, respectively) solution (1 mg/ml) was well mixed with 50 ml urea solution (200 mg/ml) to obtain the precursor solution for preparing N-doped graphene. Then, the precursor solution was reacted under hydrothermal treatment for 5 h at 180°C, and black product was obtained. Centrifugal washing was performed for several times with distilled water and ethanol. N-doped graphene was finally obtained by vacuum drying at 60°C. The samples were signed as GO-U, GO-OH-U, GO = O-U, GO-OOH-U, and GO-avg-U.

## Results and discussion

**Figure 1:**
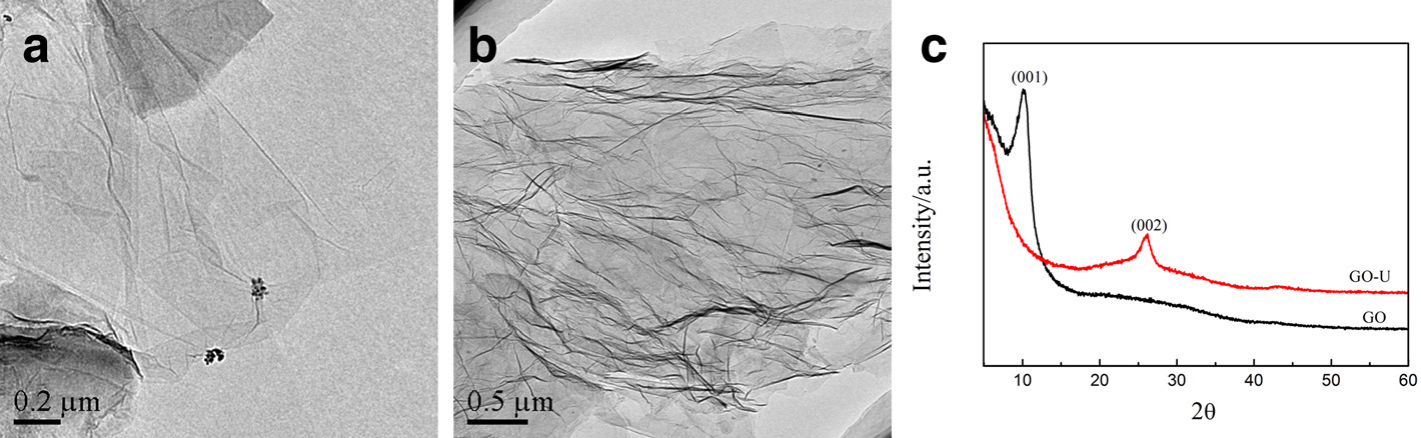
TEM image of (a) GO, (b)GO-U, and the (c) XRD pattern of the samples.

Figure 1a shows TEM image of the pristine graphene oxide obtained by Hummer’s method; it can be seen that the pristine graphene oxide is transparent and displays gauze-like morphology with slight folds and crimped edges which might originate from oxygenic functional groups. The high transparency of the sample indicates few layered structure and well dispersion of the graphene oxide layers. The thickness of the sample can be determined by AFM (Additional file 1: Figure S1). The results show that the graphene oxide layer has uniform thickness of around 1.9 nm with 2 to 3 layers [23]. After nitrogen doping, as shown in Figure 1b, the sample shows obvious wrinkled structure, indicating agglomeration of the GO layers due to defect formation after nitrogen doping within the graphite lattice, which is similar to the bamboo-shaped feature of nitrogen-doped carbon nanotubes [24].

X-ray diffraction (XRD) patterns of the graphite, pristine GO, and GO-U are displayed in Figure 1c. A sharp diffraction peak at 2*θ* = 26° can be indexed as (002) crystal plane of graphite, indicating well-ordered structure of graphite layers with spacing of about 0.34 nm. The characteristic diffraction peak of (002) crystal plane disappears completely after the graphite is fully oxidized, and a new diffraction peak at approximately 11° emerges which can be ascribed to (001) crystal plane of graphite oxide [25]. After nitrogen doping and reduction reaction, the peak at 26° reappears with broader width and weaker intensity than pristine graphite and the peak at approximately 11° disappears, implying reduction of the graphite oxide. Nevertheless, residual oxygen functional groups, defects produced by the introduced nitrogen atoms, and carbon atom loss destroy the graphite crystal structure, meanwhile increase the degree of disorder within the graphene sheet. While GO could be well dispersed forming golden colloid solution in water, N-doped graphene was insoluble in water as suspended black solid. These experimental phenomena conform well to the XRD analysis.

**Figure 2:**
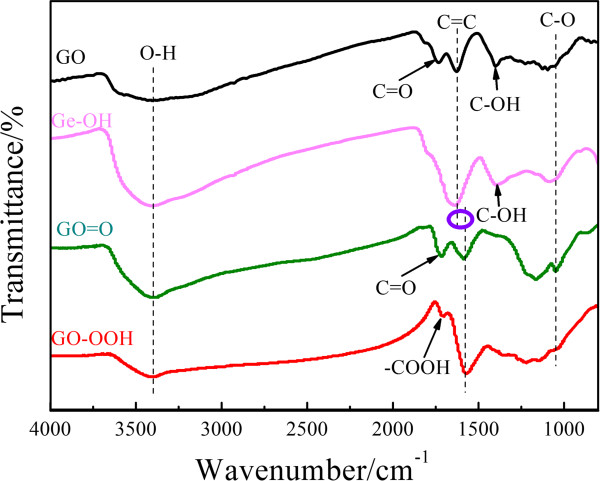
FT-IR spectra of GO, GO-OH, GO = O, and GO-OOH.

Figure 2 shows Fourier transform infrared (FTIR) spectra of GO functionalized with different oxygenic groups. Peaks at 3,406, 1,733, 1,628, 1,399, and 1,095 cm^-1^ can be ascribed to stretching vibrations of O-H, C = O, C = C, C-OH, and C-O bonds [26], respectively. By performing hydroxyl, carbonyl, and carboxyl reaction on the GO, oxygenic functional groups on the graphene surface are altered accordingly. For the Ge-OH sample, it retains its characteristic peaks for C = C and C-OH, and the characteristic peaks for O-H and C-O at 1,095 and 3,406 cm^-1^ are strengthened; whereas, the characteristic peak of C = O disappears. Compared with those of Ge-OH, the characteristic peak intensity of the C = O bond of GO = O sample (1,720 cm^-1^) evidently increases and the intensity of the characteristic peak for C-OH decreases. Sample GO-OOH exhibits weak characteristic peaks of -COOH, C-OH, and C-O at 1,706, 1,399, and 1,095 cm^-1^, respectively. Compared to GO and Ge-OH, a red-shift phenomenon is observed clearly in the peak representing C = C bonds of GO = O and GO-OOH samples (approximately 1,580 cm^-1^). This red-shift may be attributed to the abundance of C = O structures in GO = O and GO-OOH; these highly electronegative groups weaken the charge density of carbon atoms in the sample skeleton. Additional file 1: Figure S2 in the support information shows electron spectroscopy determination of the samples which is consistent with the FT-IR data.

Previous literature reported that C1s peak of GO can usually be divided into five sub-peaks [27]: C-C (284.5 ± 0.1 eV), C-OH (285.4 ± 0.3 eV), C-O-C (286.6 ± 0.2 eV), C = O (287.6 ± 0.6 eV), and -COOH (288.7 ± 0.2 eV). Additional file 1: Figure S2 shows composition of GO surface with different oxygenic functional groups as well as their carbon distributions. Different types of oxidized graphene (e.g., carboxyl graphene) are obtained after the treatment using GO as the precursor as shown in Table 1.It can be determined that the graphene surface is functionalized by different oxygenic groups after different surface modification. For the Ge-OH sample, the dominated hydroxyl groups reach 40.0% of the oxygenic groups; carbonyl groups on the GO = O sample is up to 22.8%; the GO-OOH sample owns 37.3% carboxyl functional groups on the graphene surface, which is consistent with the FT-IR spectra shown in Figure 2. It not only verifies successful preparation of the different functional (oxygen-containing) graphene but also provides a solid foundation for future studies on the effects of different oxygenic functional groups on nitrogen doping behavior.

**Table 1:** Elemental composition and distribution of the type of oxygenic groups on the surface of GO-,Ge-OH, GO = O, GO-COOH, and GO-avg samples

**Sample**	**C/at.%**	**O/at.%**	**C/O**	**C distribution/%**				
				**C-C (284.5 ± 0.1 eV)**	**C-OH (285.4 ± 0.3 eV)**	**C-O-C (286.6 ± 0.2 eV)**	**C = O (287.6 ± 0.6 eV)**	**-COOH (288.7 ± 0.2 eV)**
GO	61.4	38.6	1.6	44.1	7.5	27.6	20.7	
Ge-OH	69.0	31.0	2.2	48.2	40.0	8.3	12.5	
GO = O	66.8	33.2	2.0	50.2	6.6		22.8	
GO-OOH	60.3	39.7	1.5	38.1	17.5	7.1		37.3
GO-avg	59.2	40.8	1.4	36.5	20.2	19.1	13.3	10.9

**Figure 3:**
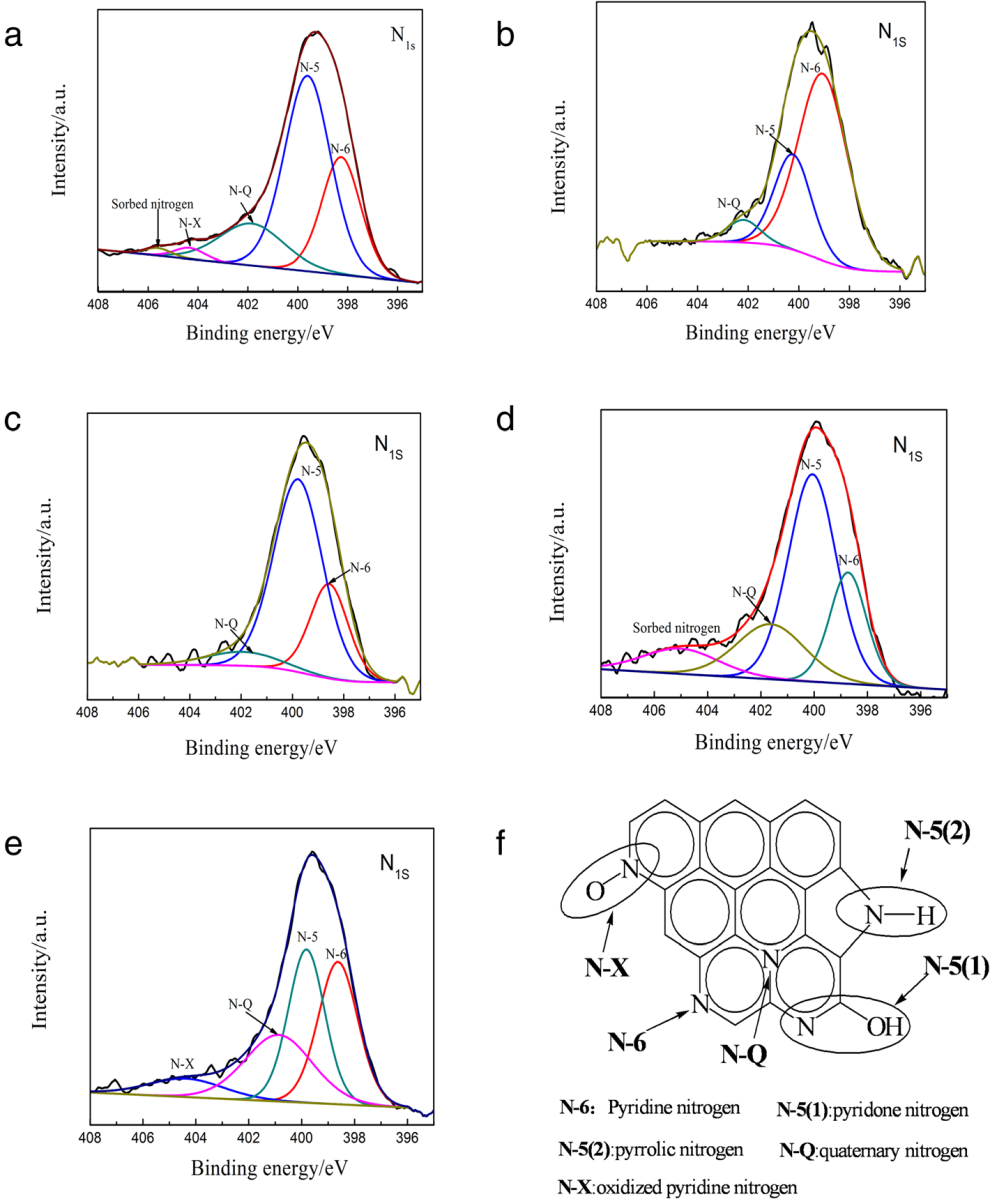
**XPS spectra of N-doped graphene and schematic illustration of nitrogen functional groups.** XPS spectra of N-doped graphene converted from GO with different oxygenic groups **(a-e)**. Schematic illustration of nitrogen functional groups in the carbon lattice **(f)**.

To better understand the influence of surface oxygenic functional groups on nitrogen doping processes, graphene oxide (GO-avg) was prepared. This sample has close amount of hydroxyl, carbonyl, and carboxyl functional groups on the graphene surface. Compared with the C/O value of GO-avg, that of GO-OOH is slightly lower while that of carbonyl graphene (GO = O) is slightly higher, which indicates that oxidation of the former increases while that of the latter decreases. By conducting a doping process on the different graphene oxides, graphene with different nitrogen contents and configurations were obtained which can be confirmed by XPS spectra analysis of N-doped graphene converted from GO with different oxygenic groups, as shown in Figure 3a-e. Figure 3f shows schematic illustration of nitrogen functional groups in the carbon lattice. Experimental results show that the oxygen content of all N-doped graphene is obviously reduced due to the N doping and the reduction process. Table 2 shows that the nitrogen content of GO-COOH-U, GO-avg-U, GO = O-U, and Ge-OH-U are 8.4, 6.1, 4.2, and 3.0 at.%, respectively, indicating that the carboxyl group evidently promotes the nitrogen doping into graphene. It should be noted that the nitrogen doping content of GO-U is 9.5 at.%, which is higher than GO-COOH. This point will be further discussed later. GO-avg-U presents the highest N-Q content (1.6 at.%) and an even distribution of various types of doped nitrogen. This result may be interpreted from the fact of the even distribution of oxygenic groups in the sample. Sample GO-COOH-U presents the highest N-5 content (5.3 at.%) and the second high N-Q content (1.4 at.%). N-6 and N-5 represent the types of doped nitrogen with the lowest contents in Ge-OH-U and GO = O-U, and the N-Q contents are significantly low with only 0.1 and 0.3 at.%.

**Table 2:** Elemental composition and distribution of the type of nitrogen-containing groups on the surface of GO-U, Ge-OH-U, GO = O-U, GO-COOH-U, and GO-avg-U samples

**Sample**	**C/at.%**	**N/at.%**	**N/C/%**	**N distribution/at.%**			
				**N-6 (398.5 ± 0.3 eV)**	**N-5 (400 ± 0.2 eV)**	**N-Q (401.5 ± 0.3 eV)**	**N-X (404.4 ± 0.3 eV)**
GO-U	77.7	9.5	12.2	2.6	5.1	1.5	0.2
Ge-OH-U	77.9	3.0	3.9	2.1	0.8	0.1	
GO = O-U	74.3	4.2	5.7	1.1	2.8	0.3	
GO-OOH-U	82.4	8.4	10.2	1.2	5.3	1.4	
GO-avg-U	78.9	6.1	7.7	2.0	2.0	1.6	0.5

**Figure 4:**
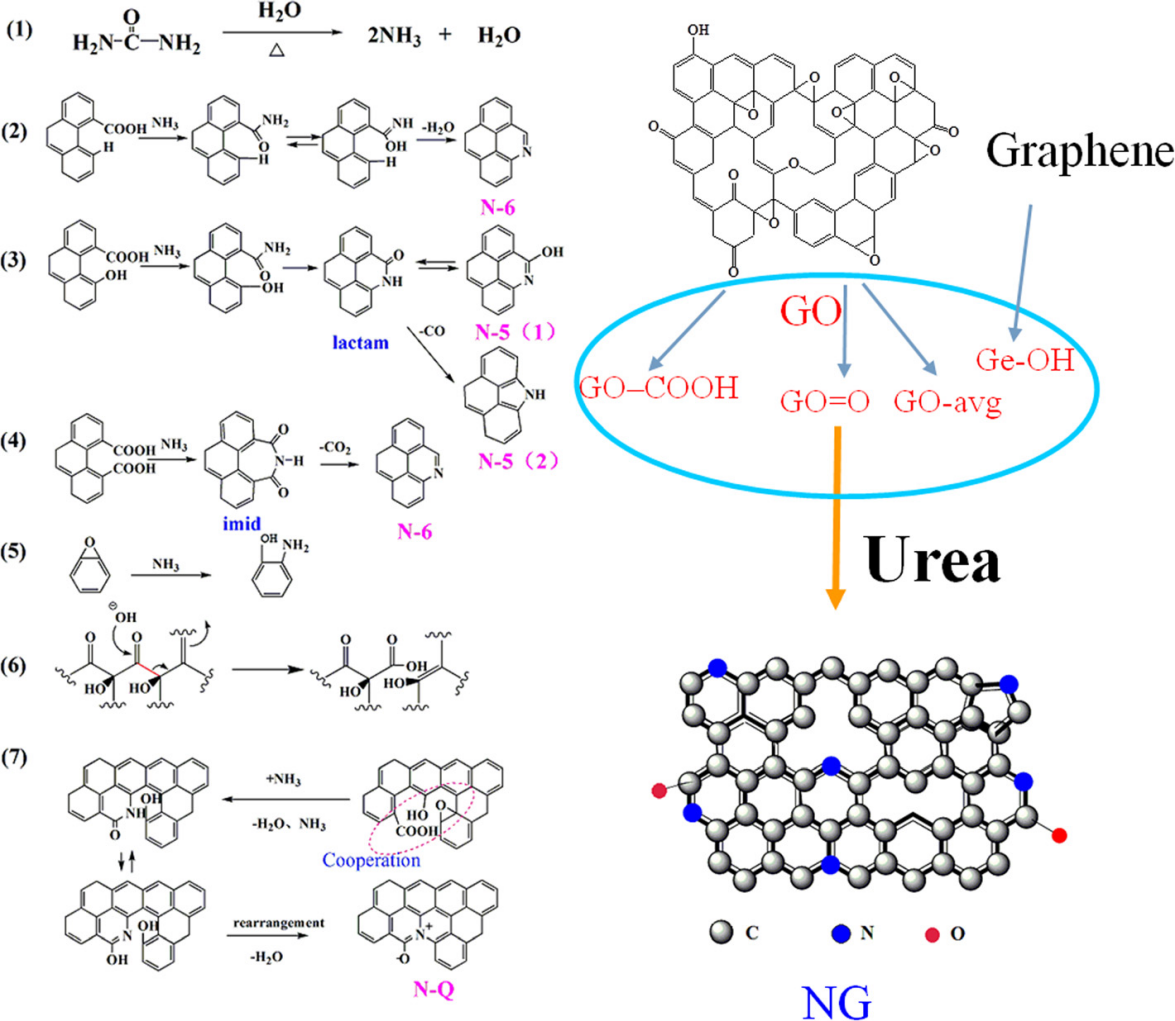
Schematic illustration of reaction pathway for the formation of N-doped graphene.

Based on the obtained data, the nitrogen doping and reduction mechanism of graphene oxide to nitrogen-doped graphene with urea under hydrothermal conditions can be proposed:

(a) Decomposition of urea under heating conditions generated H_2_O and NH_3_ (1), which acted as a nitrogen source. Under normal pressure and temperatures higher than 300°C, pyridine-like and pyrrolic nitrogen were easy to form on the graphene surface (N-5, N-6). At the same pressure with temperature over 600°C, graphitic-like nitrogen (N-Q) could be obtained [9]. Under hydrothermal conditions and temperatures lower than 200°C, N-5, N-6, and N-Q may be obtained because the sealed reactor provided an increase in pressure [8].

(b) Carboxyl groups on the graphene surface provided the main active sites for reaction with nitrogen atoms. This functional group reacted with NH_3_ to generate amides, including lactam, which was caused by dehydration, as well as imide, and initiated a series of reactions, including tautomeric transformations and removal of small molecules (e.g., CO and CO_2_), N-5 and N-6 structures (2 to 4) were formed finally [21]. Thus, more carboxyl groups on the graphene directly led to higher nitrogen-doping amount. In alkaline and high-temperature conditions, carbonyls were transformed into carboxyls (6), which reacted with NH_3_ and favored nitrogen doping [28]. This may explain why GO without carboxyl groups presented the maximum nitrogen amount after hydrothermal reduction. Moreover, the absence of contaminants and structural damages from retreatment (eg hydroxylation, carbonylation, carboxylation, etc.) can be partially related to its efficient nitrogen-doping manner.

(c) Hydroxyls were less likely to participate independently in the reactions in this system because a stable p-π conjugated structure was formed between phenolic hydroxyls and the benzene ring [29]. In addition, both long hydroxyl chain and NH_3_ are slightly alkaline. Therefore, Ge-OH yielded the lowest nitrogen doping and thought it had a large number of hydroxyl groups. After initial introduction of nitrogen atoms, the original -OH and -OH (3, 5, 7) generated from C-O-C showed their important contribution in nitrogen doping and subsequent reactions [8,10,20,29]. As for C = O, they were more likely to transform to phenols (from quinone structure) and carboxyls (just like the pathway (6) in Figure 4) to participate in the following nitrogen-doping process [30].

(d) The -OH structure that is adjacent to -COOH preferentially generated N-5 (3), while isolated -COOH and nearby -COOH structures favored the formation of N-6 (1, 4), which is identical to that for (b).

(e) The generation of the N-Q structure includes concerted reactions of various oxygenic groups (e.g., -COOH, -OH, C-O-C, etc.) (7). N-5 (2) and -OH structures are first obtained in a stepwise manner [8]. Then, dehydration and re-composition of the ring are performed to obtain N-Q. These procedures yield structures with moderate contents of various oxygenic groups, hence a higher degree of N-Q doping can be achieved.

## Conclusions

Designed synthesis of N-doped graphene with different N contents and nitrogen bonding configuration has been successfully obtained under hydrothermal conditions using urea as the nitrogen source. The experimental results indicated that different oxygenic groups (hydroxyl, carbonyl, and carboxyl) on the graphene oxide surface demonstrated different effects in the nitrogen doping process. The nitrogen doping mechanism depending on the oxygenic groups was discussed. It showed that carboxyl group played the dominant role for graphene oxide in accepting nitrogen atoms by forming N-5 and N-6 doping type, and the maximum nitrogen doping concentration was obtained in the sample. It suggested that hydroxyl did not benefit in the initial nitrogen doping, but the original -OH and C-O-C generated by the -OH played an important role in promoting nitrogen doping and subsequent reaction as long as nitrogen doping was introduced by other groups. The N-Q formation was very complex, requiring synergistic effect of various oxygenic groups. In addition, distribution and position of oxygenic groups were also non-trivial for the nitrogen doping process. We believe that this work may contribute to the understanding of the nitrogen doping mechanism on graphene and promote the development of producing novel graphene-based devices with designed surface functionalization.

## Abbreviations

TEM: transmission electron microscopy; AFM: atomic force microscopy; XRD: X-ray diffraction; XPS: X-ray photoelectron spectroscopy; FT-IR: Fourier transform infrared spectroscopy; GO: graphene oxide; N-5: pyridine-like nitrogen; N-6: pyrrolic-like nitrogen; N-Q: graphitic-like nitrogen.

## Competing interests

The authors declare that they have no competing interests.

## Authors’ contributions

YC took the tasks of data collection and analysis and draft writing. BX conducted the experiment of modifying graphene oxide by the selective oxygenic groups. YR carried out the experiment of nitrogen doping of the graphene samples. MY took the task of fabrication of graphene oxide. TX took the task of XRD data measurement. YZ designed the work, proposed growth mechanism of the graphene, and revised the manuscript. YW supervised the work and finalized the manuscript. All authors read and approved the final manuscript.

## Supplementary Material

Additional file 1: Figure S1 Typical AFM image of graphene oxide. **Figure S2.** XPS spectra of (a) GO, (b) GO-OH, (c) GO-OOH, (d) GO = O, (e) GO-avg. Click here for file
