# Supplementary material for: Designed nitrogen doping of few-layer graphene functionalized by selective oxygenic groups
Source: Nanoscale Res Lett. 2014 Nov 29;9(1):646. doi: 10.1186/1556-276X-9-646 (PMC4266512; doi:10.1186/1556-276X-9-646)
Supplement: Additional file 1: Figure S1 — Typical AFM image of graphene oxide. Figure S2. XPS spectra of (a) GO, (b) GO-OH, (c) GO-OOH, (d) GO = O, (e) GO-avg. [file 1556-276X-9-646-S1.doc]

**Additional files**


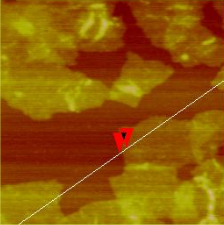

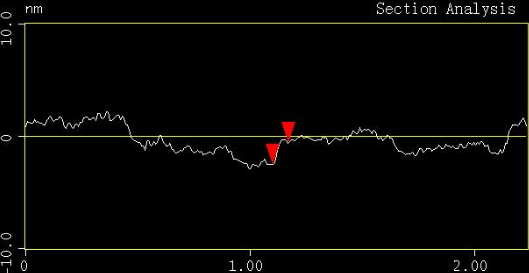


d = 1.930nm

Fig. S1 Typical AFM image of graphene oxide


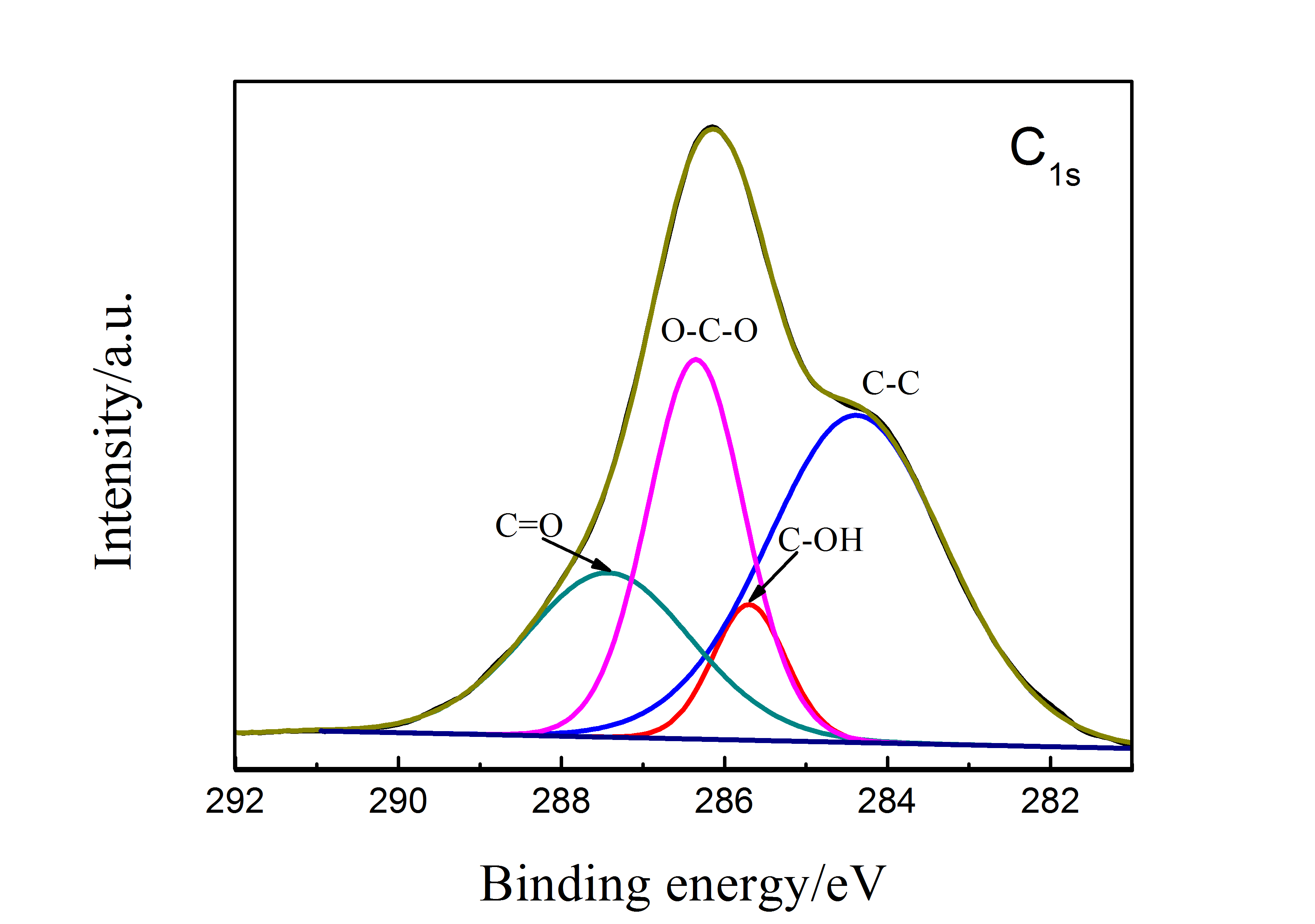

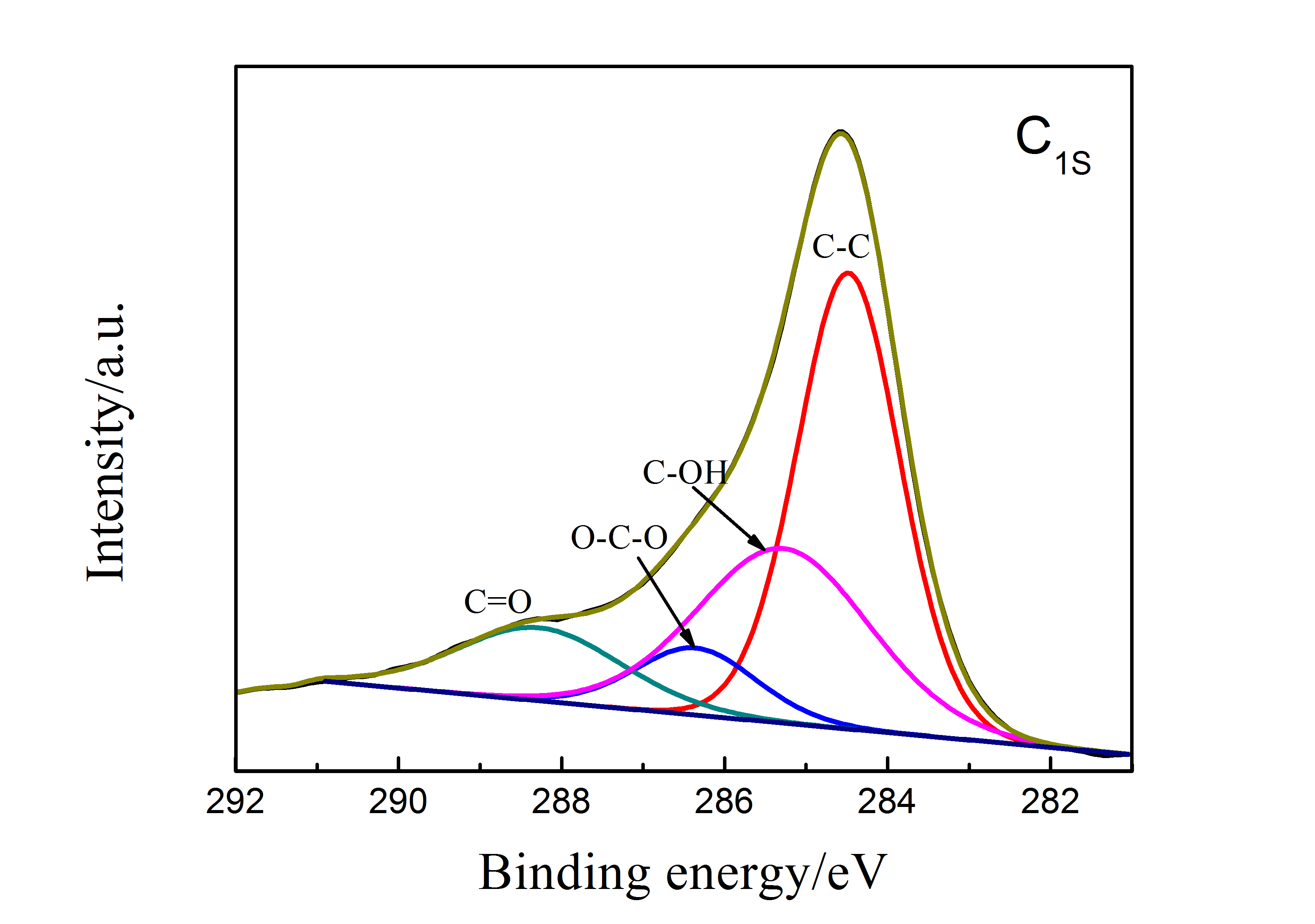

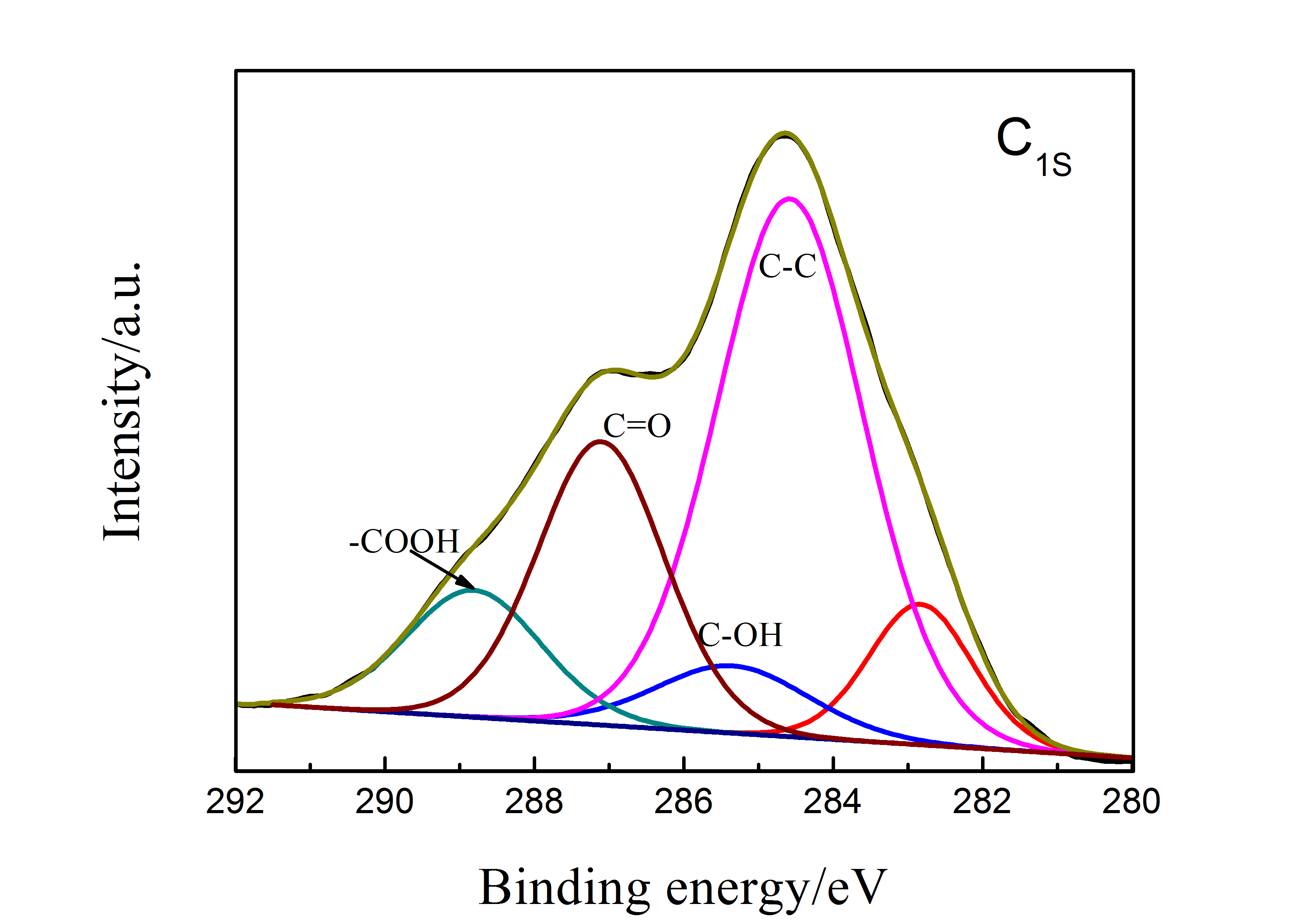

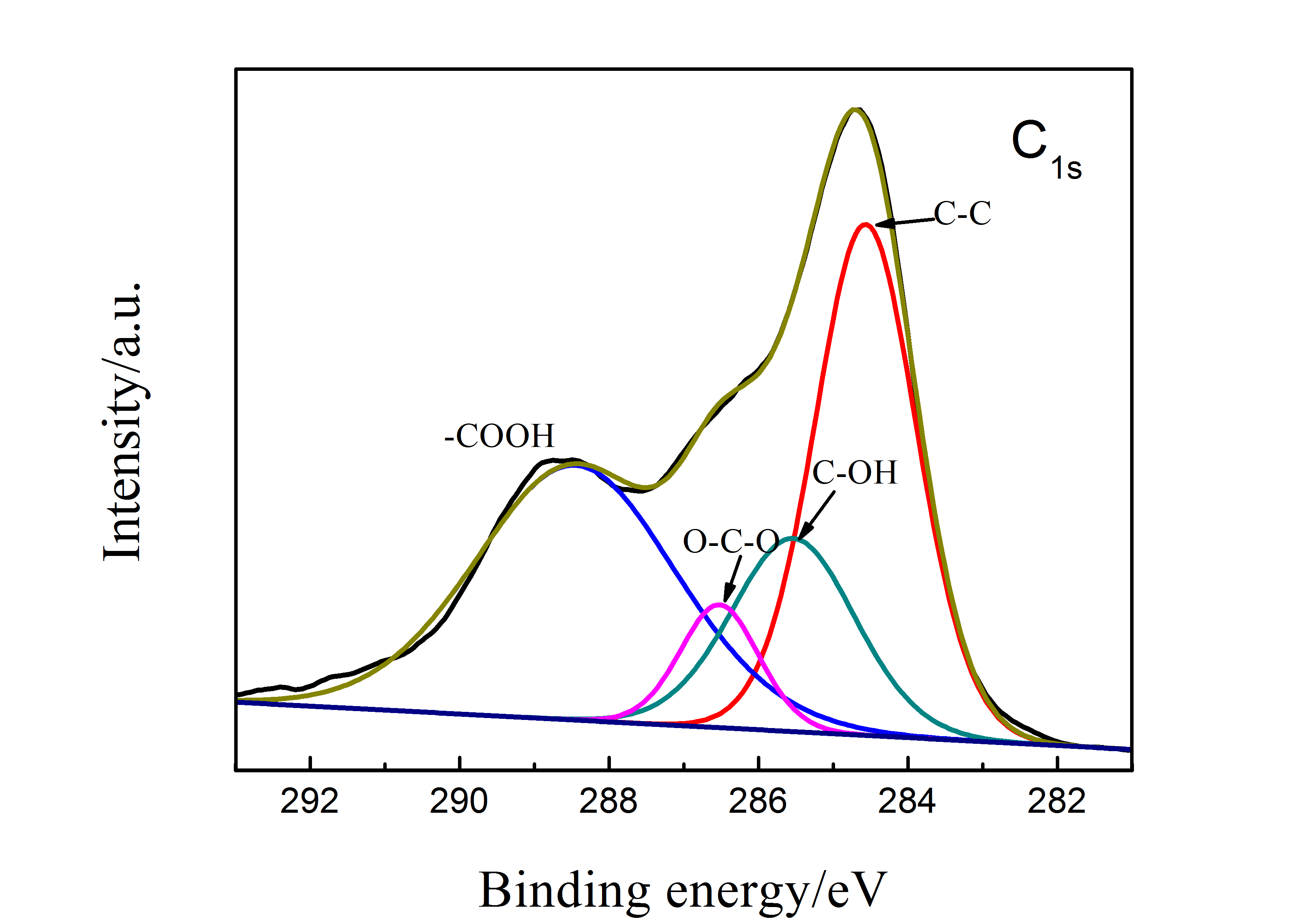

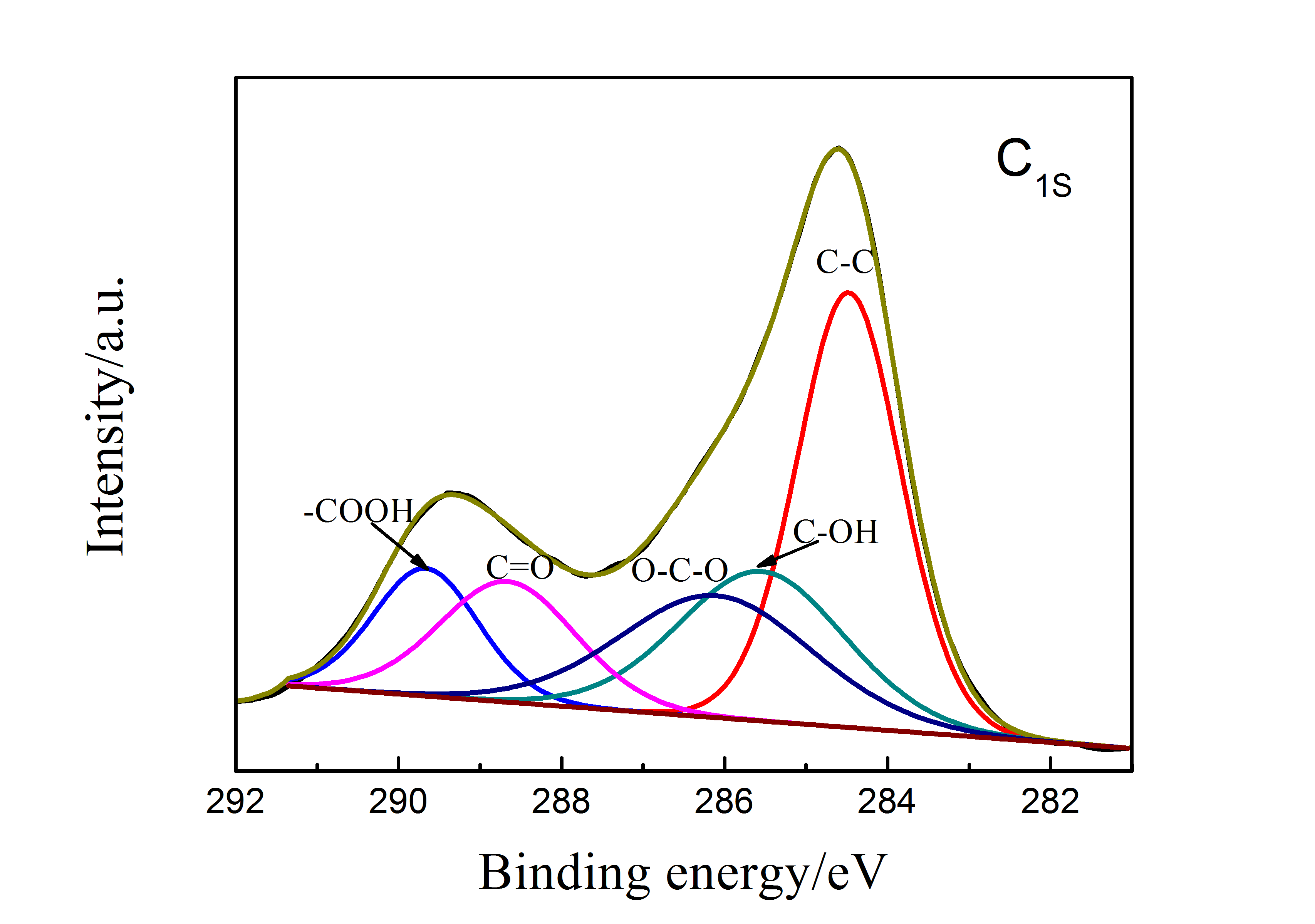


a

b

c

d

e

Fig. S2 XPS spectra of (a)GO,(b)GO-OH,(c)GO-OOH,(d)GO=O,(e)GO-avg.
